# SARS-CoV-2 omicron variant clearance delayed in breakthrough cases with elevated fasting blood glucose

**DOI:** 10.1186/s12985-022-01877-0

**Published:** 2022-09-13

**Authors:** Xiujun Zhang, Guocan Si, Huifen Lu, Wei Zhang, Shuqin Zheng, Zeyu Huang, Longgen Liu, Yuan Xue, Guojun Zheng

**Affiliations:** 1 Institute of Hepatology, The Third People’s Hospital of Changzhou, No. 300 Lanling North Road, Changzhou, 213000 Jiangsu China; 2 Department of Infectious Diseases, The Third People’s Hospital of Changzhou, Changzhou, China; 3 Clinical Laboratory, The Third People’s Hospital of Changzhou, Changzhou, China

**Keywords:** SARS-CoV-2, COVID-19, Omicron, Glucose, Uric acid

## Abstract

**Background:**

Omicron variant (B.1.1.529) is a dominant variant worldwide. However, the risk factors for Omicron variant clearance are yet unknown. The present study aimed to investigate the risk factors for early viral clearance of Omicron variant in patients with a history of inactivated vaccine injection.

**Methods:**

Demographic, clinical, and epidemiological data from 187 patients were collected retrospectively during the Omicron variant wave.

**Results:**

73/187 and 114/187 patients were administered two and three doses of vaccine, respectively. The median duration of SARS-CoV-2 RNA positivity was 9 days, and the difference between patients with two and three vaccine injections was insignificant (P = 0.722). Fever was the most common symptom (125/187), and most patients (98.4%) had a fever for < 7 days. The RNA was undetectable in 65/187 patients on day 7. Univariable logistic analysis showed that baseline glucose, uric acid, lymphocytes count, platelet count, and CD4^+^ T lymphocyte count were associated with SARS-CoV-2 RNA-positivity on day 7. Multivariable analysis showed that glucose ≥ 6.1 mmol/L and CD4^+^T lymphocytes count were independent risk factors for RNA positivity on day 7. 163/187 patients had an undetectable RNA test on day 14, and uric acid was the only independent risk factor for RNA positivity. Moreover, baseline glucose was negatively correlated with uric acid and CD4^+^ and CD8^+^ T cell count, while uric acid was positively correlated with CD4^+^ and CD8^+^ T cell count.

**Conclusions:**

Omicron variant clearance was delayed in breakthrough cases with elevated fasting blood glucose, irrespective of the doses of inactivated vaccine.

## Background

In the past 2 years, approximately 527 million patients have been infected by severe acute respiratory syndrome coronavirus 2 (SARS-CoV-2), and 6.3 million were deceased globally. Omicron variant (B.1.1.529), first reported in November 2021, has become the dominant variant worldwide [1, 2]. Although its virulence is weak, SARS-CoV-2 is more transmissible and infectious than other variants of concern [1, 3].

The spike receptor-binding domain (RBD) in the Omicron variant has enhanced binding properties with the human angiotensin-converting enzyme 2 (ACE2) receptor, which facilitates cell permeability [4]. Since RBD is the primary target of neutralizing antibodies upon vaccination or infection, whether mutations in RBD can affect the neutralizing activity of monoclonal antibodies (mAbs), leading to immune escape, is a significant concern [5, 6]. Moreover, mutations in the amino-terminal domain (NTD) also reduce the protectivity of the vaccine. Data from a multicenter retrospective study from Singapore showed that viral load was similar between vaccinated and unvaccinated patients at diagnosis. The mRNA vaccine caused a rapid decline in the Delta variant’s viral load of the Delta variant [7]. Another study showed that the viral load was lower in patients infected within 6 months of vaccination compared to the unvaccinated individuals, while no significant difference was detected in the viral load between patients vaccinated > 6 months before infection and unvaccinated individuals [8]. However, data about convalescent plasma or vaccines against the Omicron variant are disappointing [9–12]. Sera from convalescent patients infected by the Delta variant or vaccinated individuals showed low or no neutralizing activity against the Omicron variant [9, 11]. Although antibodies generated by a booster vaccine show a lower response against the Omicron variant than Delta, accumulating evidence warranted the administration of a booster vaccine dose [11, 13].

Based on previous studies, blood glucose predicted the mortality of coronavirus disease 2019 (COVID-19), even in patients without a history of diabetes [14–16]. High fasting plasma glucose level (≥ 6.1 mmol/L) at admission is an independent predictor for prolonged SARS-CoV-2 shedding [17]. Individuals with hyperglycemia had lower spike IgG titer than those with normoglycemia after two doses of the BNT162b2 vaccine, indicating that hyperglycemia is associated with a poor humoral immune response [18]. In young adults with type 1 diabetes, the elevated glucose level is positively associated with persistent symptoms [19]. The in vitro experiment confirmed that increased glucose concentration decreases the viability of A549 cells and induces ACE2 overexpression [20]. In addition, viral loads are high, and respiratory tissue damage is severe in the diabetic mice model [21].

To date, the risk factors for Omicron variant clearance are yet unknown. Herein, we investigated the risk factors for 7- and 14-day viral clearance post-infection of Omicron variant in patients with a history of inactivated vaccine injection.

## Methods

### Patients

A total of 239 patients with COVID-19 were admitted to the Third People’s Hospital of Changzhou between March 13, 2022 and May 10, 2022. All the patients were diagnosed according to the Chinese guidelines for the diagnosis and treatment of COVID-19 [22]. COVID-19 was diagnosed based on the epidemiological history and the positive RNA test in the throat swabs. Biochemical examination and a computed tomography (CT) scan were given routinely at admission. According to the guidelines for the prevention and treatment of diabetes, the disease was diagnosed based on the history of the patient, glycated hemoglobin (HbA1c), and repeated fasting glucose tests [23]. Demographic, clinical, and epidemiological data were retrospectively collected from electronic medical records and a laboratory information management system.

### Reverse transcriptase-polymerase chain reaction (RT-PCR) assay

COVID-19 was confirmed based on the RT-PCR assay performed by the Laboratory of the Third People’s Hospital of Changzhou using a commercial kit (Jiangsu Bioperfectus Technologies Co., Taizhou, China). According to the manufacturer’s instructions, cycle threshold (Ct) values of *ORF1ab* and *N* genes < 35 indicated a positive RNA test. Duplicate tests were performed at 24 h if the result was negative (Ct ≥ 35).

### Statistical analysis

All analyses were performed using SPSS 25.0 software (Armonk, NY, USA). Data were expressed as median (interquartile range, IQR) or frequencies and compared using the Kruskal–Wallis test or chi-square test. Correlation analysis was performed using Spearman’s correlation test. Logistic regression analysis was conducted to identify the independent risk factors for viral clearance. A two-sided P-value < 0.05 was considered statistically significant.

## Results

### Baseline characteristics of patients with COVID-19

Among the 239 patients with COVID-19, 52 were excluded because of the undefined history of the vaccine. Data from 187 patients were analyzed, including 73 patients who had two doses of vaccine injection and 114 patients who received three doses. The duration from the first, second, and third vaccine injections to infection was defined as T1, T2, and T3, respectively. Only 5 patients had a T2 < 6 months, and none of the patients had a T3 < 1 month.

For comorbidity, 3 patients had chronic hepatitis B, 8 had diabetes, 17 had hypertension, 1 had pulmonary tuberculosis, and 1 had chronic kidney disease. None of the patients was administered glucocorticoid or immunosuppressor at admission or during hospitalization.

A total of 43 patients had elevated fasting blood glucose (≥ 6.1 mmol/L) at admission, among whom 7 patients had a diabetes history, and 1 patient was diagnosed with diabetes during hospitalization. Among the 35 patients who were not diagnosed with diabetes, the fasting blood glucose in 17 patients decreased to a normal level in a week without any hypoglycemic treatment, and 18 patients with slightly elevated glucose (≥ 6.1 mmol/L and < 7.5 mmol/L) did not undergo a repeat test. In addition, only 9 patients had a glucose ≥ 7.9 mmol/L at admission, and 4 patients had a normal glucose level in a week and were excluded from diabetes.

Fever was the most common symptom (125/187, 66.8%), and most patients (123/125, 98.4%) had a fever duration of < 7 days. Other symptoms included cough (30/187, 16.0%), pharyngalgia (22/187, 11.8%), muscular soreness (9/187, 4.8%), headache (9/187, 3.2%), and diarrhea (6/187, 3.2%). According to CT scans, 13 individuals were diagnosed with unilateral pneumonia and 6 with bilateral pneumonia, while none developed severe pneumonia or ARDS. The median duration of SARS-CoV-2 RNA-positivity was 9 days, and the difference between patients with two and three doses of vaccine injection was insignificant (Z = 0.356, P = 0.722).

### Characteristics of patients with and without SARS-CoV-2 clearance on day 7

As shown in Table 1, 65 (34.8%) patients had an undetectable RNA test on day 7. No significant difference was detected in age, diabetes, T1, T2, and duration of fever between patients with and without SARS-CoV-2 clearance. Patients with viral clearance on day 7 had a lower glucose level than those with a positive RNA test (*Z* = 3.943, P < 0.01). In addition, neutrophils were higher, while the lymphocyte and platelet counts were lower in patients with a positive RNA test than those with a negative RNA test (*Z* = 2.160, 2.014, and 2.114, respectively; P < 0.05). Moreover, CD4^+^ T lymphocytes were higher in patients with viral clearance on day 7 (*Z* = 2.647, P < 0.01).

**Table 1:** Characteristics of patients with and without SARS-CoV-2 on day 7

Variables	Positive (n = 122)	Negative (n = 65)	Z or χ^2^	P value
Age, years	47.5(34–52)	43(33–50.5)	1.495	0.14
Male, n(%)	90(73.8)	53(81.5)	1.422	0.23
Diabetes, n(%)	6(4.9)	2(3.1)	0.351	0.72
T1,days	305.5(263.8–348.5)	315.0(265.5–348.0)	0.065	0.95
T2,days	275.5(233.5–310.3)	274.0(234.5–315.0)	0.031	0.98
Duration of fever, days	2.0(0–3.0)	1.0(0–2.0)	1.277	0.20
*Laboratory findings*				
ALT, U/L	20.2(15.0–33.2)	21.7(14.8–41.5)	0.555	0.58
AST, U/L	19.5(16.0–25.0)	20.0(16.5–25.5)	0.526	0.60
Glucose, mmol/L	5.4(4.8–6.3)	4.8(4.5–5.5)	3.943	< 0.01
Creatinine, µmol/L	69.3(58.1–76.9)	69.5(58.9–79.6)	0.546	0.59
Uric acid, µmol/L	304.6(259.5–363.9)	329.8(273.3–395.3)	1.806	0.07
CRP, mg/L	4.0(2.0–11.1)	4.7(1.9–9.3)	0.338	0.74
Neutrophils, E + 09/L	4.2(3.2–5.3)	3.6(2.4–5.0)	2.160	0.03
Lymphocytes, E + 09/L	1.1(0.7–1.4)	1.2(0.9–1.6)	2.014	0.04
Platelets, E + 09/L	202.0 (174.8–234.3)	224.0(188.5–257.5)	2.114	0.04
CD4^+^T	424.0(275.8–647.8)	550.0(412.0–705.5)	2.647	< 0.01
CD8^+^T	310.5(203.8–483.0)	359.0(260.5–494.0)	1.550	0.12
B cells count	123.0(79.0–233.0)	158.0(99.0–258.0)	1.963	0.05
*CT findings*				
Unilateral pneumonia, n(%)	10(8.20)	3(4.62)	0.841	0.55
Bilateral pneumonia, n(%)	4(3.28)	2(3.08)	0.006	0.99

Data were expressed as median (IQR) for continuous variables and n (%) for categorical values, and were compared using Kruskal–Wallis test or Chi-square test. T1, duration from the first infection of vaccine to diagnosis; T2, duration from the second infection of vaccine to diagnosis; ALT, alanine aminotransferase; AST, aspartate transaminase; CRP, C-reaction protein; CT, computer tomography

### Risk factors for SARS-CoV-2 RNA positivity on day 7

As shown in Table 2, the univariable logistic analysis showed that baseline glucose, uric acid, lymphocyte count, platelet count, and CD4^+^T lymphocyte count are associated with SARS-CoV-2 RNA positivity on day 7. Then, multivariable analysis showed that baseline glucose and CD4^+^T lymphocytes count were independent risk factors for SARS-CoV-2 RNA positivity on day 7 (odds ratio (OR) = 0.631 and 1.001, 95% confidence interval (CI): 0.456–0.874 and 1.000–1.002, P < 0.01 and P = 0.02, respectively).

**Table 2:** Risk factors for SARS-CoV2 clearance on day 7

Baseline variables	Univariate			Multivariate		
	Odds ratio	95%CI	*P*	Odds ratio	95%CI	*P*
Age (years)	0.979	0.955–1.005	0.11			
Sex	0.637	0.302–1.342	0.24			
T1	0.999	0.994–1.005	0.87			
T2	1.000	0.995–1.006	0.91			
ALT	1.004	0.991–1.017	0.53			
Glucose	0.607	0.433–0.851	< 0.01	0.631	0.456–0.874	< 0.01
Creatinine	1.006	0.987–1.025	0.55			
Uric acid	1.003	1.000–1.007	0.07			
Lymphocytes	1.668	1.026–2.712	0.04			
Platelets	1.006	1.000–1.011	0.04			
CD4 + T cells	1.001	1.000–1.002	< 0.01	1.001	1.000–1.002	0.02
CD8 + T cells	1.001	0.999–1.002	0.28			
B cells	1.002	1.000–1.004	0.11			

CI, 95% confidence interval; T1, duration from the first infection of vaccine to diagnosis; T2, duration from the second infection of vaccine to diagnosis; ALT, alanine aminotransferase

More patients had a glucose level ≥ 6.1 mmol/L at admission in the RNA-positive group (35/122, 28.7%) compared to those in the RNA-negative group (8/65, 12.3%) (χ^2^ = 6.426, P = 0.01). Then, the multivariable analysis used glucose ≥ 6.1 mmol/L as a categorical value, and the data showed that glucose ≥ 6.1 mmol/L and CD4^+^ T lymphocytes count were independent risk factors for SARS-CoV-2 RNA-positivity on day 7 (OR = 0.351 and 1.001, 95% CI: 0.150–0.825 and 1.000–1.002, P = 0.02 and P = 0.01, respectively).

### Characteristics of patients with and without SARS-CoV-2 clearance on day 14

As shown in Table 3, most patients had an undetectable RNA test on day 14 (163/187, 87.2%). Patients with viral clearance at day 14 had higher uric acid levels and lymphocyte counts than those with a positive RNA test (*Z* = 2.997 and 2.084, P < 0.01 and = 0.04, respectively). The CD4^+^ and CD8^+^ T cell count was high in patients with viral clearance (*Z* = 2.382 and 2.094, both P < 0.05).

**Table 3:** Characteristics of patients with and without SARS-CoV-2 on day 14

Variables	Positive (n = 24)	Negative (n = 163)	Z or χ^2^	P value
Age, years	46.5(40.0–56.5)	46.0(34.0–51.0)	1.132	0.26
Male, n (%)	17(70.8)	126(77.3)	0.486	0.49
Diabetes, n (%)	2(8.3)	6(3.7)	1.106	0.27
T1, days	290.0(258.5–321.3)	315.0(266.0–352.0)	1.737	0.08
T2, days	270.0(221.5–291.8)	275.0(234.0–317.0)	0.998	0.32
Duration of fever, days	2.0(0–4.0)	1.0(0–3.0)	0.537	0.59
*Laboratory findings*				
ALT, U/L	19.6(13.0–26.2)	21.1(15.3–37.1)	1.636	0.10
AST, U/L	19.0(16.0–22.0)	20.0(16.0–25.0)	0.670	0.50
Glucose, mmol/L	5.4(4.8–6.7)	5.1(4.7–5.9)	1.526	0.13
Creatinine, µmol/L	75.8(55.9–82.7)	69.0(58.5–76.9)	1.539	0.12
Uric acid, µmol/L	269.3(224.2–316.8)	322.4(270.3–380.2)	2.997	< 0.01
CRP, mg/L	6.8(3.4–11.1)	3.9(1.8–9.6)	1.695	0.09
Neutrophils, E + 09/L	3.6(2.9–4.6)	4.1(3.0–5.3)	1.244	0.21
Lymphocytes, E + 09/L	0.8(0.6–1.3)	1.1(0.8–1.5)	2.084	0.04
Platelets, E + 09/L	193.5(161.3–222.5)	209.0(179.0–252.0)	1.810	0.07
CD4^+^T	387.5(238.0–503.0)	482.0(332.0–693.0)	2.382	0.02
CD8^+^T	291.0(162.5–384.0)	346.0(220.0–495.0)	2.094	0.04
B cells count	11.4(7.8–16.2)	11.8(7.9–16.2)	0.215	0.83
*CT findings*				
Unilateral pneumonia, n(%)	6(25.0)	7(4.3)	13.865	< 0.01
Bilateral pneumonia, n(%)	1(4.2)	5(3.1)	0.081	0.57

Data were expressed as median (IQR) for continuous variables and n (%) for categorical values, and were compared using Kruskal–Wallis test or Chi-square test. T1, duration from the first infection of vaccine to diagnosis; T2, duration from the second infection of vaccine to diagnosis; ALT, alanine aminotransferase; AST, aspartate transaminase; CRP, C-reaction protein; CT, computer tomography

### Risk factors for SARS-CoV-2 RNA positivity on day 14

As shown in Table 4, the univariate logistic analysis showed that baseline uric acid, lymphocyte count, platelet count, and CD4^+^ and CD8^+^ T lymphocyte count were associated with SARS-CoV-2 RNA-positivity on day 14. Then, multivariate analysis showed that only baseline uric acid is the independent risk factor for SARS-CoV-2 RNA positivity on day 14 (OR = 1.008, 95% CI: 1.002–1.014, P = 0.01).

**Table 4:** Risk factors for SARS-CoV2 clearance on day 14

Baseline variables	Univariate			Multivariate		
	Odds ratio	95%CI	*P*	Odds ratio	95%CI	*P*
Age (years)	0.973	0.938–1.009	0.14			
Sex	0.713	0.275–1.850	0.49			
T1	1.007	0.998–1.016	0.12			
T2	1.004	0.997–1.012	0.27			
ALT	1.015	0.990–1.042	0.24			
Glucose	0.899	0.734–1.101	0.30			
Creatinine	0.980	0.956–1.005	0.11			
Uric acid	1.008	1.002–1.014	0.01	1.008	1.002–1.014	0.01
Lymphocytes	2.677	0.986–7.266	0.05			
Platelet	1.009	1.000–1.018	0.05			
CD4^+^T cells	1.002	1.000–1.004	0.04			
CD8^+^T cells	1.003	1.000–1.006	0.03			
B cells	1.003	0.999–1.007	0.20			

CI, 95% confidence interval; T1, duration from the first infection of vaccine to diagnosis; T2, duration from the second infection of vaccine to diagnosis; ALT, alanine aminotransferase

Adjusted multivariable analysis was conducted when CD4^+^ and CD8^+^ T lymphocyte count was deleted, and data showed that uric acid is the only independent risk factor for SARS-CoV-2 RNA positivity on day 14.

### ***Correlation between glucose, uric acid, CD4***^+^***T, and CD8***^+^***T cell count***

**Fig. 1:**
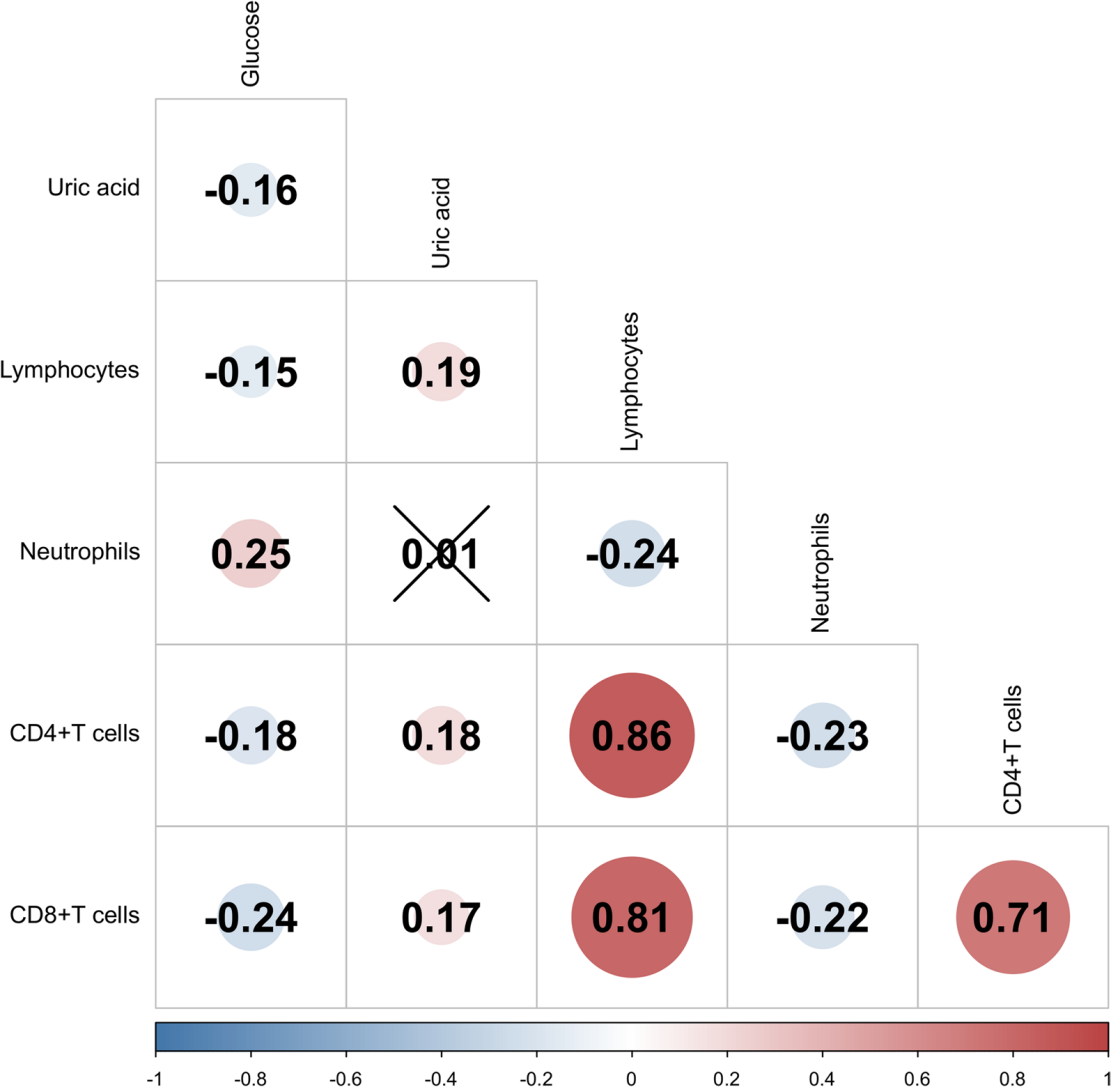
Correlation between serum glucose, uric acid, CD4^+^T and CD8^+^T cells count in Omicron variant infected patients

As shown in Fig. 1, baseline glucose was negative with uric acid, lymphocyte count, CD4^+^ T cell count, and CD8^+^ T cell count (all P < 0.05), while uric acid was positively correlated with CD4^+^ T cell and CD8^+^ T cell count (both P < 0.05). In addition, there was a positive correlation between CD4^+^ T and CD8^+^ T cell count (r = 0.710, P < 0.01).

## Discussion

In the present study, we investigated the risk factors for early clearance of Omicron variant in patients with a history of inactivated vaccine injection. On day 7 after admission, 34.8% of patients had an undetectable RNA test, and baseline glucose ≥ 6.1 mmol/L and CD4^+^ T lymphocyte count were independent risk factors for SARS-CoV-2 RNA positivity. On day 14 after admission, SARS-CoV-2 RNA became undetectable in 87.2% of patients, and uric acid was the only independent risk factor for RNA-positivity.

The knowledge of prolonged SARS-CoV-2 shedding during Omicron waves is limited, especially for patients receiving inactivated vaccine injections. For most mAbs, the neutralizing activity against the Omicron variant is limited [10, 11, 24]. Wang et al. reported that the seroconversion rate of neutralizing antibodies was just 3.3% for individuals who received two doses of inactivated vaccine, while the rate was 95% for those who received three doses of vaccine [25]. Nonetheless, the neutralizing antibody titer induced by three vaccine doses was 16.5-fold low for the Omicron variant [25]. In the present study, the duration of SARS-CoV-2 shedding was similar between patients who received two and three doses of the vaccine. Thus, it can be speculated that mutations in RBD affect the neutralizing activity of antibodies induced by the inactivated vaccine. Since most patients were vaccinated > 6 months before infection, the long interval from vaccination to infection may account for the non-optimal protection activity. These results suggest that effective vaccine strategies are warranted.

Several studies indicated that glucose at admission is an independent risk factor for the poor prognosis of COVID-19 during the Delta variant wave [15, 16, 20]. Moreover, high glucose at admission is an independent predictor for prolonged SARS-CoV-2 shedding, irrespective of a history of diabetes [17]. Similar results were obtained in the present study during the Omicron variant wave. Glucose-like metabolite deficiency may facilitate viral entry and replication, as described previously [21]. Another mechanism is that the production of neutralizing antibodies is reduced in diabetes [18, 26]. In addition, our results showed that CD4^+^ T cell count is low in patients without early viral clearance and is negatively related to glucose. Thus, glucose and cellular immunity regulation may be an appropriate strategy for managing COVID-19.

The present data showed that uric acid is the only independent predictor for SARS-CoV-2-positivity on day 14, negatively correlated with glucose, and positively correlated with CD4^+^ and CD8^+^ T cells. Interestingly, the patients with lower serum uric acid levels experienced severe symptoms than those with higher uric acid levels [27, 28]. SARS-CoV-2 induces low serum uric acid [28] level and causes hyperglycemia in a cat model [29]. The interactions between uric acid, glucose, impaired cellular or humoral immunity, and viral replication are yet to be elucidated. Although diabetes is detected in only a few cases in the present study, elevated fasting blood glucose was a common phenomenon at admission; however, the underlying mechanisms are yet unknown. Moreover, the effect of hypoglycemic agents, such as sodium-glucose cotransporter 2 (SGLT2) inhibitors, on COVID-19 remains an interesting issue worth exploration [30].

The main limitation of the present study is that neutralizing antibodies at admission are not estimated. Regarding the Omicron variant spread, data from the present study highlighted the potential predictive value of metabolic factors, such as glucose and uric acid, in clinical practice. Typically, the benefits of the vaccine outweigh the potential risks [31, 32]; hence, vaccination during the pandemic of the Omicron variant should be administered without hesitation. Since outbreaks of COVID-19 occurred in different cities, a multicenter study with large sample size is warranted. Another limitation is that only 3 patients received HbA1c tests; otherwise, sufficient data could have been collected.

## Conclusion

Omicron variant clearance was delayed in breakthrough cases with elevated fasting blood glucose, regardless of the inactivated vaccine doses.

## Data Availability

Derived data are available on reasonable request.

## Abbreviations

SARS-CoV-2: Severe acute respiratory syndrome coronavirus 2
RBD: Receptor-binding domain
ACE2: Angiotensin-converting enzyme 2
mAbs: Monoclonal antibodies
NTD: Amino-terminal domain
COVID-19: Coronavirus disease 2019
RT-PCR: Reverse transcriptase-polymerase chain reaction
Ct: Cycle threshold
IQR: Interquartile range
CT: Computed tomography
OR: Odds ratio
CI: Confidence interval
